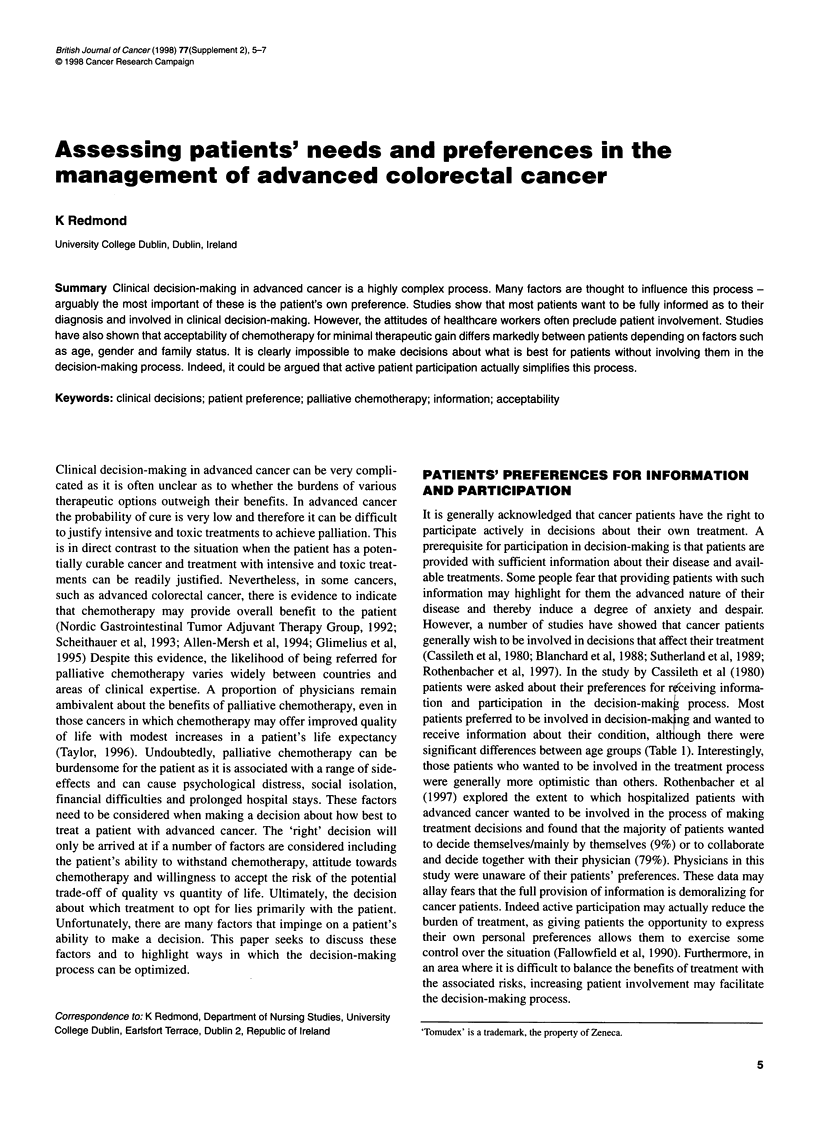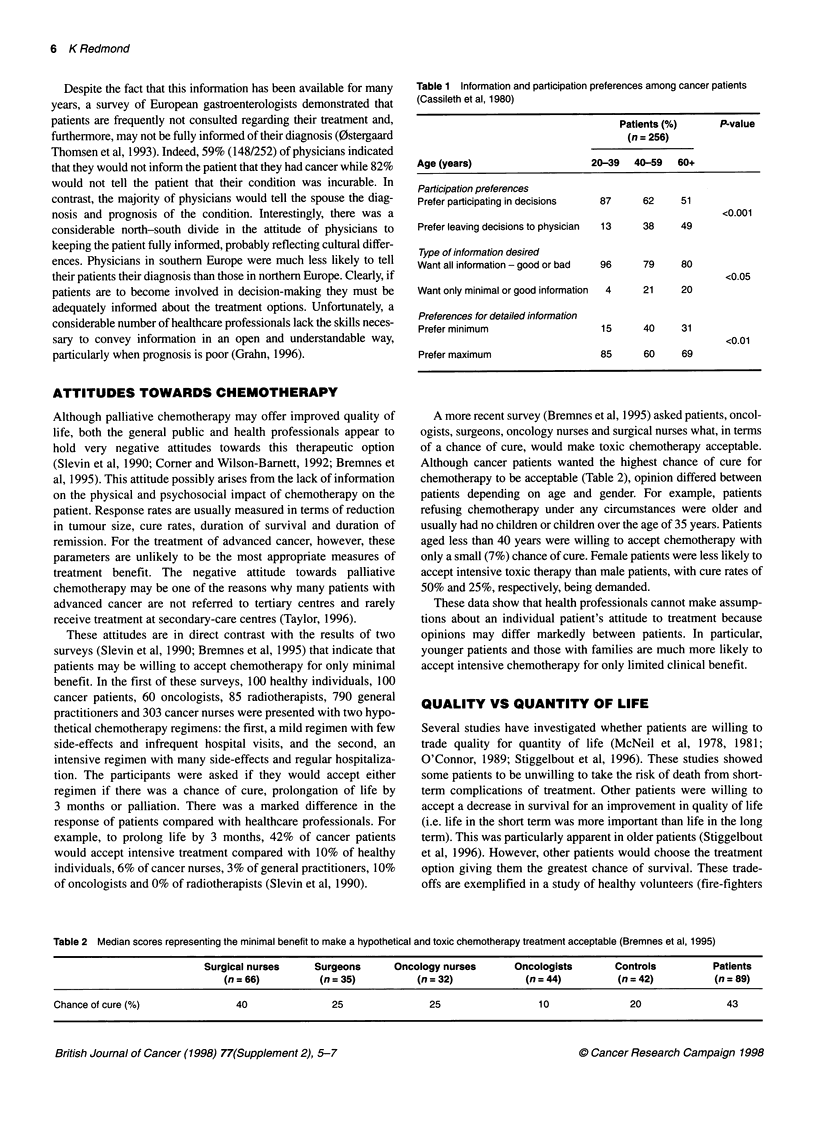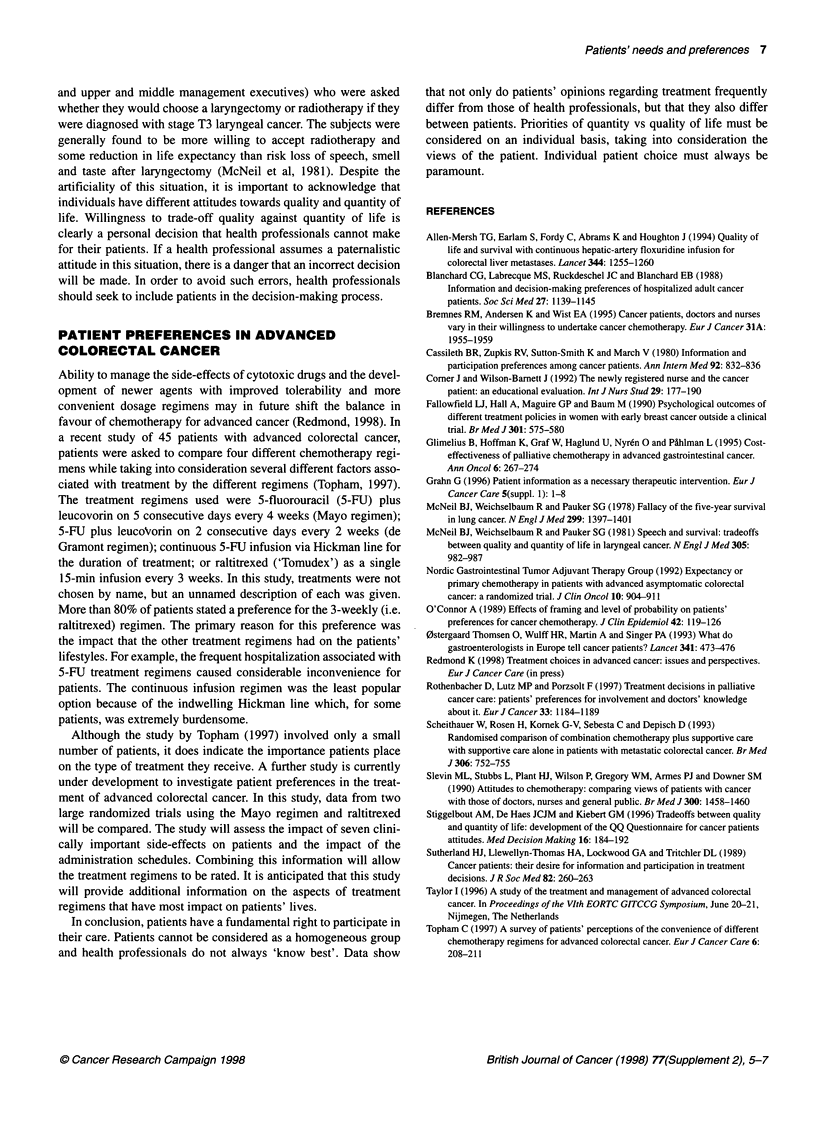# Assessing patients' needs and preferences in the management of advanced colorectal cancer.

**DOI:** 10.1038/bjc.1998.419

**Published:** 1998

**Authors:** K. Redmond

**Affiliations:** University College Dublin, Republic of Ireland.

## Abstract

Clinical decision-making in advanced cancer is a highly complex process. Many factors are thought to influence this process arguably the most important of these is the patient's own preference. Studies show that most patients want to be fully informed as to their diagnosis and involved in clinical decision-making. However, the attitudes of healthcare workers often preclude patient involvement. Studies have also shown that acceptability of chemotherapy for minimal therapeutic gain differs markedly between patients depending on factors such as age, gender and family status. It is clearly impossible to make decisions about what is best for patients without involving them in the decision-making process. Indeed, it could be argued that active patient participation actually simplifies this process.


*British Journal of Cancer* (1998) **77**(Supplement 2), 5–7
© 1998 Cancer Research Campaign

### Assessing patients' needs and preferences in the management of advanced colorectal cancer

**K Redmond**

University College Dublin, Dublin, Ireland

**Summary** Clinical decision-making in advanced cancer is a highly complex process. Many factors are thought to influence this process – arguably the most important of these is the patient's own preference. Studies show that most patients want to be fully informed as to their diagnosis and involved in clinical decision-making. However, the attitudes of healthcare workers often preclude patient involvement. Studies have also shown that acceptability of chemotherapy for minimal therapeutic gain differs markedly between patients depending on factors such as age, gender and family status. It is clearly impossible to make decisions about what is best for patients without involving them in the decision-making process. Indeed, it could be argued that active patient participation actually simplifies this process.

**Keywords:** clinical decisions; patient preference; palliative chemotherapy; information; acceptability

Clinical decision-making in advanced cancer can be very complicated as it is often unclear as to whether the burdens of various therapeutic options outweigh their benefits. In advanced cancer the probability of cure is very low and therefore it can be difficult to justify intensive and toxic treatments to achieve palliation. This is in direct contrast to the situation when the patient has a potentially curable cancer and treatment with intensive and toxic treatments can be readily justified. Nevertheless, in some cancers, such as advanced colorectal cancer, there is evidence to indicate that chemotherapy may provide overall benefit to the patient (Nordic Gastrointestinal Tumor Adjuvant Therapy Group, 1992; Scheithauer et al, 1993; Allen-Mersh et al, 1994; Glimelius et al, 1995) Despite this evidence, the likelihood of being referred for palliative chemotherapy varies widely between countries and areas of clinical expertise. A proportion of physicians remain ambivalent about the benefits of palliative chemotherapy, even in those cancers in which chemotherapy may offer improved quality of life with modest increases in a patient's life expectancy (Taylor, 1996). Undoubtedly, palliative chemotherapy can be burdensome for the patient as it is associated with a range of side-effects and can cause psychological distress, social isolation, financial difficulties and prolonged hospital stays. These factors need to be considered when making a decision about how best to treat a patient with advanced cancer. The 'right' decision will only be arrived at if a number of factors are considered including the patient's ability to withstand chemotherapy, attitude towards chemotherapy and willingness to accept the risk of the potential trade-off of quality vs quantity of life. Ultimately, the decision about which treatment to opt for lies primarily with the patient. Unfortunately, there are many factors that impinge on a patient's ability to make a decision. This paper seeks to discuss these factors and to highlight ways in which the decision-making process can be optimized.

*Correspondence to:* K Redmond, Department of Nursing Studies, University College Dublin, Earlsfort Terrace, Dublin 2, Republic of Ireland

### PATIENTS' PREFERENCES FOR INFORMATION AND PARTICIPATION

It is generally acknowledged that cancer patients have the right to participate actively in decisions about their own treatment. A prerequisite for participation in decision-making is that patients are provided with sufficient information about their disease and available treatments. Some people fear that providing patients with such information may highlight for them the advanced nature of their disease and thereby induce a degree of anxiety and despair. However, a number of studies have showed that cancer patients generally wish to be involved in decisions that affect their treatment (Cassileth et al, 1980; Blanchard et al, 1988; Sutherland et al, 1989; Rothenbacher et al, 1997). In the study by Cassileth et al (1980) patients were asked about their preferences for receiving information and participation in the decision-making process. Most patients preferred to be involved in decision-making and wanted to receive information about their condition, although there were significant differences between age groups (Table 1). Interestingly, those patients who wanted to be involved in the treatment process were generally more optimistic than others. Rothenbacher et al (1997) explored the extent to which hospitalized patients with advanced cancer wanted to be involved in the process of making treatment decisions and found that the majority of patients wanted to decide themselves/mainly by themselves (9%) or to collaborate and decide together with their physician (79%). Physicians in this study were unaware of their patients' preferences. These data may allay fears that the full provision of information is demoralizing for cancer patients. Indeed active participation may actually reduce the burden of treatment, as giving patients the opportunity to express their own personal preferences allows them to exercise some control over the situation (Fallowfield et al, 1990). Furthermore, in an area where it is difficult to balance the benefits of treatment with the associated risks, increasing patient involvement may facilitate the decision-making process.

'Tomudex' is a trademark, the property of Zeneca.

Despite the fact that this information has been available for many years, a survey of European gastroenterologists demonstrated that patients are frequently not consulted regarding their treatment and, furthermore, may not be fully informed of their diagnosis (Østergaard Thomsen et al, 1993). Indeed, 59% (148/252) of physicians indicated that they would not inform the patient that they had cancer while 82% would not tell the patient that their condition was incurable. In contrast, the majority of physicians would tell the spouse the diagnosis and prognosis of the condition. Interestingly, there was a considerable north–south divide in the attitude of physicians to keeping the patient fully informed, probably reflecting cultural differences. Physicians in southern Europe were much less likely to tell their patients their diagnosis than those in northern Europe. Clearly, if patients are to become involved in decision-making they must be adequately informed about the treatment options. Unfortunately, a considerable number of healthcare professionals lack the skills necessary to convey information in an open and understandable way, particularly when prognosis is poor (Grahn, 1996).

**Table 1** Information and participation preferences among cancer patients (Cassileth et al, 1980)

| | Patients (%) ($n$ = 256) | | | *P*-value |
|---|---|---|---|---|
| **Age (years)** | **20–39** | **40–59** | **60+** | |
| *Participation preferences* | | | | |
| Prefer participating in decisions | 87 | 62 | 51 | <0.001 |
| Prefer leaving decisions to physician | 13 | 38 | 49 | |
| *Type of information desired* | | | | |
| Want all information – good or bad | 96 | 79 | 80 | <0.05 |
| Want only minimal or good information | 4 | 21 | 20 | |
| *Preferences for detailed information* | | | | |
| Prefer minimum | 15 | 40 | 31 | <0.01 |
| Prefer maximum | 85 | 60 | 69 | |

### ATTITUDES TOWARDS CHEMOTHERAPY

Although palliative chemotherapy may offer improved quality of life, both the general public and health professionals appear to hold very negative attitudes towards this therapeutic option (Slevin et al, 1990; Corner and Wilson-Barnett, 1992; Bremnes et al, 1995). This attitude possibly arises from the lack of information on the physical and psychosocial impact of chemotherapy on the patient. Response rates are usually measured in terms of reduction in tumour size, cure rates, duration of survival and duration of remission. For the treatment of advanced cancer, however, these parameters are unlikely to be the most appropriate measures of treatment benefit. The negative attitude towards palliative chemotherapy may be one of the reasons why many patients with advanced cancer are not referred to tertiary centres and rarely receive treatment at secondary-care centres (Taylor, 1996).

These attitudes are in direct contrast with the results of two surveys (Slevin et al, 1990; Bremnes et al, 1995) that indicate that patients may be willing to accept chemotherapy for only minimal benefit. In the first of these surveys, 100 healthy individuals, 100 cancer patients, 60 oncologists, 85 radiotherapists, 790 general practitioners and 303 cancer nurses were presented with two hypothetical chemotherapy regimens: the first, a mild regimen with few side-effects and infrequent hospital visits, and the second, an intensive regimen with many side-effects and regular hospitalization. The participants were asked if they would accept either regimen if there was a chance of cure, prolongation of life by 3 months or palliation. There was a marked difference in the response of patients compared with healthcare professionals. For example, to prolong life by 3 months, 42% of cancer patients would accept intensive treatment compared with 10% of healthy individuals, 6% of cancer nurses, 3% of general practitioners, 10% of oncologists and 0% of radiotherapists (Slevin et al, 1990).

A more recent survey (Bremnes et al, 1995) asked patients, oncologists, surgeons, oncology nurses and surgical nurses what, in terms of a chance of cure, would make toxic chemotherapy acceptable. Although cancer patients wanted the highest chance of cure for chemotherapy to be acceptable (Table 2), opinion differed between patients depending on age and gender. For example, patients refusing chemotherapy under any circumstances were older and usually had no children or children over the age of 35 years. Patients aged less than 40 years were willing to accept chemotherapy with only a small (7%) chance of cure. Female patients were less likely to accept intensive toxic therapy than male patients, with cure rates of 50% and 25%, respectively, being demanded.

These data show that health professionals cannot make assumptions about an individual patient's attitude to treatment because opinions may differ markedly between patients. In particular, younger patients and those with families are much more likely to accept intensive chemotherapy for only limited clinical benefit.

### QUALITY VS QUANTITY OF LIFE

Several studies have investigated whether patients are willing to trade quality for quantity of life (McNeil et al, 1978, 1981; O'Connor, 1989; Stiggelbout et al, 1996). These studies showed some patients to be unwilling to take the risk of death from short-term complications of treatment. Other patients were willing to accept a decrease in survival for an improvement in quality of life (i.e. life in the short term was more important than life in the long term). This was particularly apparent in older patients (Stiggelbout et al, 1996). However, other patients would choose the treatment option giving them the greatest chance of survival. These trade-offs are exemplified in a study of healthy volunteers (fire-fighters

**Table 2** Median scores representing the minimal benefit to make a hypothetical and toxic chemotherapy treatment acceptable (Bremnes et al, 1995)

| | Surgical nurses ($n$ = 66) | Surgeons ($n$ = 35) | Oncology nurses ($n$ = 32) | Oncologists ($n$ = 44) | Controls ($n$ = 42) | Patients ($n$ = 89) |
|---|---|---|---|---|---|---|
| Chance of cure (%) | 40 | 25 | 25 | 10 | 20 | 43 |

© Cancer Research Campaign 1998

and upper and middle management executives) who were asked whether they would choose a laryngectomy or radiotherapy if they were diagnosed with stage T3 laryngeal cancer. The subjects were generally found to be more willing to accept radiotherapy and some reduction in life expectancy than risk loss of speech, smell and taste after laryngectomy (McNeil et al, 1981). Despite the artificiality of this situation, it is important to acknowledge that individuals have different attitudes towards quality and quantity of life. Willingness to trade-off quality against quantity of life is clearly a personal decision that health professionals cannot make for their patients. If a health professional assumes a paternalistic attitude in this situation, there is a danger that an incorrect decision will be made. In order to avoid such errors, health professionals should seek to include patients in the decision-making process.

### PATIENT PREFERENCES IN ADVANCED COLORECTAL CANCER

Ability to manage the side-effects of cytotoxic drugs and the development of newer agents with improved tolerability and more convenient dosage regimens may in future shift the balance in favour of chemotherapy for advanced cancer (Redmond, 1998). In a recent study of 45 patients with advanced colorectal cancer, patients were asked to compare four different chemotherapy regimens while taking into consideration several different factors associated with treatment by the different regimens (Topham, 1997). The treatment regimens used were 5-fluorouracil (5-FU) plus leucovorin on 5 consecutive days every 4 weeks (Mayo regimen); 5-FU plus leucovorin on 2 consecutive days every 2 weeks (de Gramont regimen); continuous 5-FU infusion via Hickman line for the duration of treatment; or raltitrexed ('Tomudex') as a single 15-min infusion every 3 weeks. In this study, treatments were not chosen by name, but an unnamed description of each was given. More than 80% of patients stated a preference for the 3-weekly (i.e. raltitrexed) regimen. The primary reason for this preference was the impact that the other treatment regimens had on the patients' lifestyles. For example, the frequent hospitalization associated with 5-FU treatment regimens caused considerable inconvenience for patients. The continuous infusion regimen was the least popular option because of the indwelling Hickman line which, for some patients, was extremely burdensome.

Although the study by Topham (1997) involved only a small number of patients, it does indicate the importance patients place on the type of treatment they receive. A further study is currently under development to investigate patient preferences in the treatment of advanced colorectal cancer. In this study, data from two large randomized trials using the Mayo regimen and raltitrexed will be compared. The study will assess the impact of seven clinically important side-effects on patients and the impact of the administration schedules. Combining this information will allow the treatment regimens to be rated. It is anticipated that this study will provide additional information on the aspects of treatment regimens that have most impact on patients' lives.

In conclusion, patients have a fundamental right to participate in their care. Patients cannot be considered as a homogeneous group and health professionals do not always 'know best'. Data show that not only do patients' opinions regarding treatment frequently differ from those of health professionals, but that they also differ between patients. Priorities of quantity vs quality of life must be considered on an individual basis, taking into consideration the views of the patient. Individual patient choice must always be paramount.

### REFERENCES

Allen-Mersh TG, Earlam S, Fordy C, Abrams K and Houghton J (1994) Quality of life and survival with continuous hepatic-artery floxuridine infusion for colorectal liver metastases. *Lancet* **344**: 1255–1260

Blanchard CG, Labrecque MS, Ruckdeschel JC and Blanchard EB (1988) Information and decision-making preferences of hospitalized adult cancer patients. *Soc Sci Med* **27**: 1139–1145

Bremnes RM, Andersen K and Wist EA (1995) Cancer patients, doctors and nurses vary in their willingness to undertake cancer chemotherapy. *Eur J Cancer* **31A**: 1955–1959

Cassileth BR, Zupkis RV, Sutton-Smith K and March V (1980) Information and participation preferences among cancer patients. *Ann Intern Med* **92**: 832–836

Corner J and Wilson-Barnett J (1992) The newly registered nurse and the cancer patient: an educational evaluation. *Int J Nurs Stud* **29**: 177–190

Fallowfield LJ, Hall A, Maguire GP and Baum M (1990) Psychological outcomes of different treatment policies in women with early breast cancer outside a clinical trial. *Br Med J* **301**: 575–580

Glimelius B, Hoffman K, Graf W, Haglund U, Nyrén O and Påhlman L (1995) Cost-effectiveness of palliative chemotherapy in advanced gastrointestinal cancer. *Ann Oncol* **6**: 267–274

Grahn G (1996) Patient information as a necessary therapeutic intervention. *Eur J Cancer Care* **5**(suppl. 1): 1–8

McNeil BJ, Weichselbaum R and Pauker SG (1978) Fallacy of the five-year survival in lung cancer. *N Engl J Med* **299**: 1397–1401

McNeil BJ, Weichselbaum R and Pauker SG (1981) Speech and survival: tradeoffs between quality and quantity of life in laryngeal cancer. *N Engl J Med* **305**: 982–987

Nordic Gastrointestinal Tumor Adjuvant Therapy Group (1992) Expectancy or primary chemotherapy in patients with advanced asymptomatic colorectal cancer: a randomized trial. *J Clin Oncol* **10**: 904–911

O'Connor A (1989) Effects of framing and level of probability on patients' preferences for cancer chemotherapy. *J Clin Epidemiol* **42**: 119–126

Østergaard Thomsen O, Wulff HR, Martin A and Singer PA (1993) What do gastroenterologists in Europe tell cancer patients? *Lancet* **341**: 473–476

Redmond K (1998) Treatment choices in advanced cancer: issues and perspectives. *Eur J Cancer Care* (in press)

Rothenbacher D, Lutz MP and Porzsolt F (1997) Treatment decisions in palliative cancer care: patients' preferences for involvement and doctors' knowledge about it. *Eur J Cancer* **33**: 1184–1189

Scheithauer W, Rosen H, Kornek G-V, Sebesta C and Depisch D (1993) Randomised comparison of combination chemotherapy plus supportive care with supportive care alone in patients with metastatic colorectal cancer. *Br Med J* **306**: 752–755

Slevin ML, Stubbs L, Plant HJ, Wilson P, Gregory WM, Armes PJ and Downer SM (1990) Attitudes to chemotherapy: comparing views of patients with cancer with those of doctors, nurses and general public. *Br Med J* **300**: 1458–1460

Stiggelbout AM, De Haes JCJM and Kiebert GM (1996) Tradeoffs between quality and quantity of life: development of the QQ Questionnaire for cancer patients attitudes. *Med Decision Making* **16**: 184–192

Sutherland HJ, Llewellyn-Thomas HA, Lockwood GA and Tritchler DL (1989) Cancer patients: their desire for information and participation in treatment decisions. *J R Soc Med* **82**: 260–263

Taylor I (1996) A study of the treatment and management of advanced colorectal cancer. In *Proceedings of the VIth EORTC GITCCG Symposium*, June 20–21, Nijmegen, The Netherlands

Topham C (1997) A survey of patients' perceptions of the convenience of different chemotherapy regimens for advanced colorectal cancer. *Eur J Cancer Care* **6**: 208–211

© Cancer Research Campaign 1998